# Impact of the Prolymphangiogenic Crosstalk in the Tumor Microenvironment on Lymphatic Cancer Metastasis

**DOI:** 10.1155/2014/639058

**Published:** 2014-09-01

**Authors:** Simona L. Schlereth, Nasrin Refaian, Sandra Iden, Claus Cursiefen, Ludwig M. Heindl

**Affiliations:** ^1^Department of Ophthalmology, University of Cologne, Kerpener Straße 62, 50937 Cologne, Germany; ^2^Cologne Excellence Cluster on Cellular Stress Responses in Aging-Associated Diseases (CECAD) and Center for Molecular Medicine Cologne (CMMC), University of Cologne, Joseph-Stelzmann-Straße 26, 50931 Cologne, Germany

## Abstract

Lymphangiogenesis is a very early step in lymphatic metastasis. It is regulated and promoted not only by the tumor cells themselves, but also by cells of the tumor microenvironment, including cancer associated fibroblasts, mesenchymal stem cells, dendritic cells, or macrophages. Even the extracellular matrix as well as cytokines and growth factors are involved in the process of lymphangiogenesis and metastasis. The cellular and noncellular components influence each other and can be influenced by the tumor cells. The knowledge about mechanisms behind lymphangiogenesis in the tumor microenvironmental crosstalk is growing and offers starting points for new therapeutic approaches.

## 1. Introduction

The spread of tumor cells via the lymphogen route into the draining lymph nodes is common in many malignant tumors, including malignant melanoma of the skin [1], head and neck squamous cell carcinoma [2], squamous cell carcinoma of the uterine cervix [3], colorectal carcinoma [4], breast cancer [5], and malignant melanoma of the conjunctiva [6–13]. Sentinel lymph node biopsy allows early detection of micrometastasis resulting in staging and treatment changes.

The outgrowth of new lymphatic vessels from preexisting lymphatic vessels (lymphangiogenesis) has recently gained much interest in tumor research since it is the initial step in lymphogenic metastasis [14]. Although the role of intratumoral versus peritumoral lymphangiogenesis is still debated, its role as a decisive risk factor for tumor metastasis is now established.

Lymphangiogenesis is mediated by binding of the lymphangiogenic growth factors vascular endothelial growth factor- (VEGF-) C and VEGF-D to their specific lymphatic receptor, VEGF receptor 3 [15]. VEGF-C and VEGF-D can be released by a variety of tumor cells or by peritumoral nonmalignant cells of the tumor microenvironment [16–19], thus explaining the occurrence of tumor-associated lymphangiogenesis.

The cellular crosstalk in the tumor microenvironment is likely to play a role in promoting lymphangiogenesis and thus lymphatic metastasis. A variety of factors in the tumor microenvironment, including extracellular matrix (ECM) with cancer-associated fibroblasts (CAFs) and mesenchymal stem cells (MSCs), cells of the innate and adaptive immune system (dendritic cells, macrophages, and T- and B-cells) as well as cytokines and growth factors produced by the tumor and stromal cells [20, 21], has been considered to contribute to this process.

This review focuses on the role of tumor microenvironmental components in tumor-associated lymphangiogenesis and therefore the lymphatic metastasis cascade. Better understanding of these mechanisms is required to improve future therapeutic strategies aiming at minimizing the lymphatic spread of the tumor to the regional lymph nodes in order to the prolong survival of cancer patients.

## 2. Cytokines and Growth Factors Control Lymphangiogenesis

Growth factors of the vascular endothelial growth factor (VEGF) family are well understood in lymphangiogenesis. VEGF is the target of one of the first therapeutics: VEGF blocking antibody bevacizumab is used in colon cancer [22].

VEGF-D has been shown to induce the formation of blood and lymphatic vessels in tumors and VEGF-D expression on tumor cells led to increased lymphatic metastasis [23]. However, other authors emphasize the tissue specific effects on blood or lymph endothelial growth of VEGF-D [24]. In many forms of human cancer, a correlation of VEGF-C expression within the primary tumor and lymph node metastasis has been observed [25–30]. VEGF-C overexpression in breast cancer increased intratumoral lymphangiogenesis and was associated with enhanced metastasis into draining lymph nodes and lungs [31]. This might be caused by a tumor secreted VEGF-C dependent increase of matrix metalloproteinase- (MMP-) 9 production, followed by an increased matrix degradation and migration [32]. Other studies conclude that tumor derived VEGF-C draining to the regional lymph nodes may promote the outgrowth of lymph node metastasis [33].

Controversy exists whether VEGF-A is able to induce lymphangiogenesis. Recent studies indicate that the VEGF-A/VEGF-R2 signaling pathway is involved in lymphangiogenesis [14, 34]. Hirakawa et al. detected that VEGF-A overexpressing primary tumors can induce lymph node lymphangiogenesis and were associated with increased lymph node metastasis [35]. Lymph node lymphangiogenesis per se is thought to actively promote metastasis [36] and can also be induced by tumor cells [37].

Beside the VEGF family, the angiopoietins- (Ang-) 1 and Ang-2 are important in tumor angiogenesis. They bind to their receptors Tie 1 and Tie 2 on vascular endothelial cells and are involved in lymphangiogenesis and metastasis [38–42]. Ang-2 is upregulated by different factors including VEGF-A or insulin like growth factor 1 and induces angiogenesis in the presence of VEGF-A [39]. A reduced prognosis has been shown for different tumors overexpressing Ang-2 [39]. Ang-2 seems to have a destabilizing effect on blood vessels, an early step in neovascularization [43], whereas Ang-1 expressed by pericytes and others promotes stability of vessels [38]. In pancreatic cancer, elevated circulating Ang-2 was correlated with the extent of lymphatic metastasis and therefore seems to participate in the control of lymphatic metastasis [44].

Other factors that are involved in lymphangiogenesis are platelet derived growth factor- (PDGF-) BB [45], fibroblast growth factor- (FGF-) 2 [46], sphingosine 1 phosphate (S1P) [47], and hepatocyte growth factor (HGF) [48].

Lymphatic endothelium cells express different markers, including lymphatic vessel endothelial hyaluronan receptor-1 (LYVE-1), podoplanin D2-40, prospero homeobox transcription factor 1 (prox1), and VEGF-R3 [49]. Lately, besides the significant correlation of lymphatic markers LYVE-1 and podoplanin D2-40 [50] in many forms of cancer and their negative correlation to prognosis mentioned above, prox1 and forkhead box (FOX) C2, regulators of angiogenesis and lymphangiogenesis, came into focus of cancer research. Sasahira et al. report that prox1 expression correlated with progression, lymphatic vessel density, metastasis, and worse prognosis [51]. Prox1 activated VEGF-C expression in vitro, whereas FOXC2 enhances prox1 and VEGF-A expression [51].

Chemokines are important signal proteins, involved in cell migration and chemotaxis. Chemokine ligands bind to their specific receptors. Metastatic cells seem to adopt this mechanism and express analogue receptors to improve their migration to distinct tissues [52]. Many different chemokine pathways are known and their role in cancer has been completely and well reviewed by others [53]; therefore, in this review we focus on chemokine axes involved in lymphatic metastasis: the chemokine receptor 7 (CCR7) with its ligands CCL19 and 21 and the CXCR4/CXCR12 axis. The CCR7 axis is a very important physiological axis for migration of immune cells and CCL21 regulates the homing to the lymphoid tissues [54].

Chemokines have been shown to be involved in tumor lymphangiogenesis and metastasis; for example, VEGF-C upregulated chemokine ligand 21 (CCL21) on lymphatic endothelium, whereby CCR7 expressing tumor cells were attracted towards the lymphatic vessels [32].

Many studies show that primary tumor cells and metastatic cells express CCR7 in the draining lymph node and that there is a significant correlation between lymph node metastasis and CCR7 expression in many tumor entities [55–58]. In one study the authors suggest that CCR7 enhances metastasis by upregulating MMP-9 expression [59]. Li et al. showed that hypoxia may induce CCR7 expression on tumor cells to stimulate migration and invasion of lung cancer cells, using the HIF1α and HIF2α pathway [60].

Other chemokines such as CXC chemokine type 2 (CXCR2) seem to be involved in lymphangiogenesis, as a high expression of CXCR2 is associated with increased lymph node metastases and a reduced prognosis in resected esophageal carcinoma [61].

Chemokine receptor CXCR4 is involved in metastasis of multiple cancer entities, including breast cancer [62], gastric cancer [63], prostate cancer [64], melanoma [65], uveal melanoma [66], or glioblastoma [67], to name just a few.

CXCR4 is upregulated in metastatic breast cancer cell lines and lymph node metastasis [62], and cells expressing CXCR4 predominantly migrate to tissues that express the ligand CXCL12 [62]. These tissues include the common sides of breast cancer metastasis, including lung, lymph node, brain, and bone marrow [5, 62]. Interestingly, in vivo inhibition of the CXCR4/CXCL12 axis reduced lymph node and lung metastasis [62]. Others showed that the de novo expression of CXCR4 is sufficient for metastasis to occur, shown by the B16 melanoma cell line transfected with CXCR4 [68]. In gastric cancer, CXCR4 expression is involved in lymph node metastasis [63, 69].

In prostate cancer, CXCR4 expression has been shown to increase tumor invasion and metastasis [64]. It may therefore serve as a prognostic marker in prostate cancer [70].

One last example is the CXCR3-CXCL9 axis: CXCR3 expression has been detected in several human melanoma cell lines and the mouse melanoma cell line B16F10. The loss of CXCR3 expression reduces lymph node metastasis in a murine melanoma model [71].

**Figure 1:**
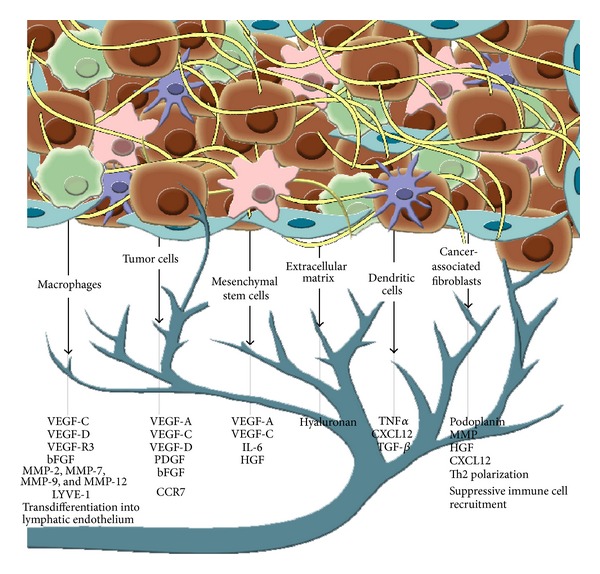
The prolymphangiogenic crosstalk of the tumor microenvironment: tumor cells as well as macrophages, dendritic cells, the extracellular matrix, cancer-associated fibroblasts, and mesenchymal stem cells can promote lymphangiogenesis by secretion or expression of different factors.

To summarize, the best-studied group of growth factors is the VEGF family, whereby in many forms of cancer an association between VEGF-C and metastasis has been recognized. Within the chemokines in cancer, the CXCR4/CXCL12 axis is currently best characterized and CXCR4 is a ubiquitously expressed receptor on tumor cells. Chemokine expression is often associated with elevated lymphatic metastasis. Tumor cells seem to have adopted these migration paths for facilitated access into lymphatic vessels and towards the draining lymph nodes. Cytokines and growth factors involved in lymphangiogenesis are summarized in Figure 1 and explained in detail in the following sections.

## 3. Senescence and Senescence-Associated Secretory Phenotype: Cell Autonomous and Nonautonomous Roles

Most mammalian cells have a limited proliferative capacity, and after various rounds of proliferation accompanied by telomere shortening, cells undergo permanent cell cycle arrest and enter a state called cellular senescence. Senescent cells remain viable and metabolic active and thereby further contribute to tissue homeostasis. Senescence can be prematurely induced by stress factors and DNA damage, for example, upon oncogene expression or UV irradiation, and is mediated by activation of the Arf/p53/p21 and/or p16/pRb pathways [72]. Senescence has been observed in various cell types of the tumor microenvironment including fibroblasts and immune cells and is considered a physiological tumor-suppressive mechanism in human cancers as it counteracts proliferation of premalignant cells [73, 74]. Human nevi, for instance, are frequently positive for activating BRafV600E mutations; however, these cells bear a senescent phenotype [75]. Abrogation of such oncogene-induced senescence by PI3K activation allows for melanoma formation [76]. Induction of cellular senescence therefore has been recognized as promising therapeutic approach to prevent the proliferation of cancer cells. Recently, however, it became apparent that senescence in surrounding tissue cells might have both tumor-suppressive as well as promoting consequences [77]. Senescent cells secrete a variety of growth and regeneration promoting cytokines, chemokines, growth factors, and proteases, a phenomenon called senescence-associated secretory phenotype (SASP). SASP has recently been described for a variety of cancers and is considered to significantly modulate the properties of the specific tumor microenvironment. SASP factors can induce recruitment of immune cells like NK-cells and T-cells that help eliminate premalignant cells, and such NK-cell recruitment appears to be critical for tumor regression in vivo [78]. On the contrary, senescent tissue cells via SASP factors can directly promote tumor cell proliferation, invasion, and immune-editing to escape elimination by the immune system, thus overall providing a tumor-permissive micromilieu. Importantly, induction of senescence in NK cells has recently been reported to promote vascular remodeling and angiogenesis [79], opening the possibility that senescence and SASP may also contribute to tumor-associated lymphangiogenesis, although this remains to be demonstrated experimentally.

In the case of SASP of senescent fibroblasts in the tumor microenvironment, a significant overlap of its expression profile with that of cancer-associated fibroblasts (CAFs, see below) has been reported. For instance, upregulation of IL-6, IL-8, various CXCLs, and MMP-3 constitutes a common signature of CAFs and SASP [80]. Together, though induction of permanent cell cycle arrest within tumor cells is a desirable feature to suppress tumorigenesis, senescence of immune and surrounding tissue cells may have opposing outcome on tumor progression and angiogenesis, underscoring the importance of a better understanding of the cross-talk between tumor cells and their particular microenvironment.

## 4. Extracellular Matrix (ECM) with Cancer-Associated Fibroblasts (CAFs) and Mesenchymal Stem Cells (MSCs) Promote Lymphangiogenesis

Tumor-associated lymphangiogenesis may arise in the tumor microenvironment. The tumor microenvironment is mainly composed of the extracellular matrix (ECM) enriched with nonmalignant stroma cells, such as cancer-associated fibroblasts (CAFs) and mesenchymal stem cells (MSCs).

### 4.1. Extracellular Matrix (ECM)

The ECM is a complex three-dimensional network made of fibrous proteins, such as collagen and fibronectin, and nonfibrous proteins, namely, glycosaminoglycans, proteoglycans, and glycoproteins. Located between cell clusters in all tissues, it strengthens the tissues, provides a channel for communication and migration within the tissue and under physiologic conditions, and acts as scaffold to keep growth factors insoluble [81–83]. Cancer cells may stimulate the tumor microenvironment by producing growth factors, including PDGF, transforming growth factor- (TGF-) *β*, VEGF, basic fibroblast growth factor (bFGF), and interleukins [83]. The altered expression of such mediators by tumor cells, which also have autocrine effects, often leads to production of proteolytic enzymes by the tumor cells [84, 85]. They may also stimulate stromal cells, for example, fibroblasts, to secrete molecules with a similar proteolytic effect on the ECM [86].

Therefore, not only tumor cells, but also stroma cell activation may modify the ECM towards an environment that promotes microinvasion of tumor cells [87]. Major components digesting ECM and cell surface proteins include MMPs, bone morphogenic protein 1 (BMP1), tissue serine proteinases, and adamalysin-related membrane proteinases [88]. Remodeling the ECM can significantly modulate migratory and angiogenic properties, for instance by release of cryptic protein sites and specific new molecule fragments [81]. Cryptic protein domains in ECM components such as fibronectin are typically masked in a folded structure and are thereby not accessible. Proteolytic enzymes can release these domains and open new integrin binding sites and antiangiogenic sequences [81] or activate latent TGF-*β* by proteolytic cleavage [89].

Hyaluronan, an important mucopolysaccharide of the ECM, provides an environment of proliferation and migration [90]. Interestingly, lymphatic endothelial cells exclusively express a hyaluronan receptor, known as LYVE-1 [91]. The functional impact of LYVE-1 receptors on tumor-associated lymphangiogenesis is not fully understood. However, it was demonstrated recently that low molecular weight hyaluronan promoted lymphatic endothelial cell (LEC) proliferation, migration, and tube formation, mediated via binding to LYVE-1 [92]. Therefore, in the tumor context hyaluronan seems to promote hem- and lymphangiogenesis.

In summary, dynamic remodeling of the ECM and cell-substrate interactions display one important feature in tumor-mediated lymphangiogenesis.

### 4.2. Cancer-Associated Fibroblasts (CAFs)

Recently, there is increasing evidence that fibroblasts are a prominent modifier of cancer progression [93, 94]. CAFs are tall spindle shaped mesenchymal cells that share characteristics with smooth muscle cells and fibroblasts [83]. They can immunohistochemically be identified with a combination of different markers, since they show an elevated expression of α-smooth muscle actin, vimentin, desmin, and fibroblast-activating protein (FAP) compared to normal stromal fibroblasts [95, 96]. CAFs can promote tumor growth and progression but also influence the stromal microenvironment by producing large amounts of growth factors, cytokines and extracellular matrix proteins (e.g., collagen and fibronectin), and MMPs [21, 97, 98]. Bauer et al. showed an increased expression of TGF-*β*2, insulin-like growth factor-binding protein 2, tumor necrosis factor (ligand) superfamily member 4, and heparin-binding EGF-like growth factor in CAFs compared to regular fibroblasts [99]. Moreover, CAFs secrete growth factors such as HGF or TGF-*β* but also ECM glycoproteins such as Tenascin-C (TNC) [100]. Tumor cells on the other hand secrete TGF-*β* or platelet-derived growth factor (PDGF), which are important factors for interactions between tumor cells and fibroblasts. TGF-*β* modulates fibroblasts and myofibroblasts towards CAFs [101, 102]. CAFs support tumor growth and metastasis indirectly through recruitment of immune cells such as tumor-associated macrophages (TAMs), myeloid suppressor cells (MDSCs), or regulatory T-cells (T_regs_). All these cells are influencing the tumor microenvironment towards an immune suppressive environment and are thereby protecting the tumor. In vivo experiments showed that elimination of CAFs favors a Th1 over a Th2 polarization in the tumor microenvironment of a murine breast cancer model [103].

The modulation of the tumor microenvironment induces angiogenesis and lymphangiogenesis. CAFs secrete the stromal cell-derived factor 1 (SDF1), also known as CX chemokine ligand 12 (CXCL12), which is important in recruitment of endothelial progenitor cells (EPCs) in tumors. CXCL12 itself stimulates the tumor growth directly, via the CXC-chemokine receptor 4 (CXCR4), expressed among others on human breast carcinoma cells [104]. An elevated amount of CAFs significantly correlated with an increased lymphatic vessel density in ovarian cancer [105]. Another study showed that CAFs express podoplanin in the context of different tumors. The podoplanin expression of CAFs positively correlated with the VEGF-C expression of the tumor cell and the intratumoral amount of CD31+ blood vessels. In contrast, the increased expression of podoplanin in CAFs negatively correlated with peritumoral microvessels and LYVE-1 positive lymphatic vessels. The expression of podoplanin in CAFs did not correlate with the VEGF-A or VEGF-D expression in tumor cells [106].

Summarizing, CAFs seem to be important in the tumor microenvironment, where they indirectly contribute to lymphangiogenesis and metastasis by the induction of Th2 T-cells, recruitment of suppressive immune cells, and secretion of growth factors. However, the exact mechanisms are not fully understood.

### 4.3. Mesenchymal Stem Cells (MSCs)

MSCs are nonhematopoietic multipotent cells that are able to differentiate into bone, fat, or cartilage tissue. They are involved in tissue repair and maintenance and have a tropism to wounded tissue [107]. In the context of trauma or tumor, they are capable of migrating towards these tissue sides, induced by chemokines or inflammatory factors [108]. MSCs show a specific migration to growth factors such as PDGF, EGF, and VEGF and a reduced migration in the presence of specific inhibitors, such as Glivec, Erbitux, and Avastin [109]. (More to therapeutic approaches is listed below; see point 5.) MSC themselves produce an amount of tumor promoting factors, including IL-6 [86], TGF-*β*, VEGF, and HGF [107, 110].

Using these factors, MSCs are capable of enhancing lymphangiogenesis and lymphatic metastasis. LECs express an HGF receptor (also known as c-Met or MET) and HGF promotes lymphatic vessel function and formation [111]. In vitro cocultures of MSCs and endothelial progenitor cells (EPCs) revealed that MSCs secreted VEGF-A in bioavailable amounts (350 pg/mL), despite the secretion of VEGF inhibitors (sVEGF-R1/sVEGF-R2) by EPCs [110]. Moreover, in a syngeneic mouse model, subcutaneous coinjection of MSCs and EPCs in Matrigel induced both blood- and lymphangiogenesis [110], highlighting the proangiogenic effect of MSCs in vivo.

VEGF-A can induce proliferation and migration of lymphatic endothelial cells (LECs). Dellinger and Brekken showed that VEGF-R2 acts as the primary receptor controlling VEGF-A induced lymphangiogenesis in an ERK1/2 and Akt-dependent manner [112]. In inflammatory neovascularization, VEGF-A stimulates LECs and lymphangiogenesis indirectly via macrophage recruitment [18].

As mentioned above, MSCs are capable of secreting IL-6. IL-6 and a proinflammatory cytokine is upregulated in different cancer entities. For example, a significant correlation between IL-6 protein and VEGF-C mRNA with lymph node metastasis in human oral squamous cell carcinoma has been demonstrated [113]. Moreover, in vitro experiments revealed that IL-6 induces VEGF-C expression in human oral squamous cell carcinoma cell line [113] and VEGF-C expression in IL-6 treated murine LECs [114].

MSCs are able to express a lymphatic phenotype, when cultured in lymphatic induced medium and VEGF-C [115]. Vice versa, tumor cells secrete growth factors, cytokines, and chemokines to promote the migration and survival of MSCs [108]. Karnoub et al. reported that MSC infiltration into tumor stroma promotes metastasis in breast cancer [116].

In conclusion, MSCs seem to have direct and indirect effect on lymphangiogenesis and lymphatic metastasis, mainly via VEGF-A, VEGF-C, HGF, and IL-6.

## 5. Immune Cells (Dendritic Cells, Macrophages) Control Lymphangiogenesis

Tumor-associated lymphangiogenesis is under the influence of innate immune cells of the tumor microenvironment, especially dendritic cells and macrophages.

### 5.1. Dendritic Cells (DCs)

DCs are the most potent antigen presenting cells of the human body. They can be subdivided into different subsets and to fully understand their functions in the tumor microenvironment, DC subsets should be examined individually with regard to influences on their behavior, dependent on different local factors. DCs are involved in tumor immunology and angiogenesis by stimulating inflammation or inducting tolerance. They can internalize tumor antigen and cross-present it to T-cells within the draining lymph node. This is an important step towards an antitumoral immune reaction [117]. However, controversial data exists on their role in the tumor microenvironment, DC activation, or tolerance induction. On the one hand, DNA derived from necrotized tumor cells may be involved in the DC activation [118]. On the other hand, tumor cells have been shown to inhibit DC maturation through the secretion of IL-10 [119]. Within DCs, two major subsets can be differentiated: the myeloid DCs (mDCs, also known as conventional DCs) and the plasmacytoid DCs (pDCs) [120]. Both can be induced towards a tumor promoting state in the tumor microenvironment: mDCs contribute to the survival of multiple melanoma cells [121]. pDCs seem to have immunoregulatory properties in the tumor microenvironment and induce T_regs_ in the human ovarian carcinoma [122]. pDCs also contribute to angiogenesis by producing proangiogenic cytokines, such as IL-8 and tumor necrosis factor alpha (TNFα) in the ovarian carcinoma [123]. pDCs were detected in solid tumor tissue and metastatic cervical lymph nodes in head and neck squamous cell carcinoma [124]. In breast cancer, pDCs infiltration into the primary tumor was associated with shorter overall survival [125].

In general, tumor-associated DCs (TADCs) can secrete different proangiogenic factors, such as TGF-*β*, granulocyte macrophage colony-stimulating factor (GM-CSF), CXCL12, or TNFα [126, 127]. TADCs are able to differentiate into endothelial-like cells under tumor specific culture conditions [128] and CD34− CD11c+ immature DCs cocultured with tumor-cell conditioned media showed an endothelial-like differentiation [129]. Whether or not DCs participate in lymphangiogenesis is still a topic of ongoing research.

DCs can be influenced by VEGFs. VEGF has been shown to inhibit DC maturation by blocking NF-*κ*B transcription [130]. In the cornea, VEGF-R3 blocking antibody reduced the DC migration towards the draining lymph nodes [131].

DCs as antigen presenting cells are able to take up antigen and migrate to the draining lymph node, guided through a CCL21 gradient. Interestingly, some tumor cells express the CCL21 receptor CCR7, thereby enabling them to access lymphatic vessels [62, 64, 132].

Regarding surface receptors of dendritic cells, programmed cell death ligand 1, PD-L1, came into focus of interest (see therapeutic approaches below). Also known as B7 homolog 1, this transmembrane protein seems to play a major role in suppressing the immune system. PD-1 and its ligand function as a complex transmit an inhibitory signal which downregulates T-cell activation and proliferation. The ligand PD-L1 is expressed on antigen presenting cells, whereas the receptor PD-1 has been found on activated T- or B-cells, macrophages, and myeloid cells as well as multiple tumor cells [133–136] and in vitro cell lines of uveal melanoma and cutaneous melanoma [137].

In breast cancer, sentinel lymph nodes with metastasis were associated with fewer mature dendritic cells within the lymph node [138]. Similarly, immature DCs have been detected in melanoma metastasis [139], but also the presence of mature DCs within the tumor tissue correlated with lymph node metastasis [125]. High mobility group box 1 (HMGB1) secreted by tumor cells induced the suppression of DCs and is associated with lymph node metastasis in human colon cancer [140]. Similarly, lymph node metastasis significantly correlated with number of DC expression in gastric cancer [141].

Recently a distinct population of DCs, namely the 6-sulfo LacNAc(+) DCs (slanDCs) were detected in metastatic tumor draining lymph nodes. Here, slanDCs surrounded the cancer cells, while being absent at the primary tumor side [142].

Taken together, DCs are able to secrete proangiogenic factors and induce an immune tolerant milieu in the tumor microenvironment. VEGF secreted by tumor cells or tumor-associated macrophages inhibits DC maturation, and a reduced number of mature dendritic cells can be associated with elevated lymph node metastasis in breast cancer [138]. However, due to their various subgroups, further studies are needed to fully understand their impact on lymphangiogenesis and metastasis.

### 5.2. Macrophages

Macrophages play an essential role in driving tumor hem- and lymphangiogenesis [143]. Known as tumor-associated macrophages (TAMs), they may sense hypoxia in tumor tissue and secrete VEGFs, basic fibroblast growth factor (bFGF), thymidine phosphorylase (TP), MMP-2, MPP-7, MPP-9 and MPP-12 [144], and urokinase type plasminogen activator (uPA) [145] to induce both hem- and lymphangiogenesis. TAMs do not only express prolymphangiogenic factors VEGF-C, VEGF-D, and VEGF-R3 [17], but they can also transdifferentiate into lymphatic endothelium [146]. TAMs have also been shown to express LYVE-1 [147, 148] and F4/80+ LYVE-1 + macrophages integrated into peritumoral lymphatic vessels [148]. TAMs are often regulated towards an M2 phenotype. In uveal melanoma, these M2 macrophages were found to be mainly CD68+ CD163+ and high amounts of these cells were associated with a poorer prognosis [149]. This observation has also been made in a variety of other tumor entities, for example, breast cancer [150], glioma [151], or melanoma [152]. In cutaneous squamous cell carcinoma, elevated VEGF-C levels derived from TAMs were associated with increased peritumoral lymphatic vessel density [153] and may thereby coordinate metastasis [154]. Depleting macrophages during tumor induction reduced incidence of ocular tumors and improved survival in mice [149, 155, 156]. VEGF-A and VEGF-C as well as MMP-9, secreted by TAMs and tumor cells, have been shown to induce peritumoral lymphangiogenesis [17, 157]. VEGF-A hereby may stimulate the upregulation of VEGF-C expression or through binding on VEGF-R2 expressed on lymphatic endothelium [42].

Other proangiogenic effects of TAMs can also be achieved indirectly, for example, via inhibition of DC maturation, and thereby contributing to an immune tolerant status [158]. An increased amount of immature DCs within tumor tissue was associated with elevated tumor vascularization [158]. This inhibitory and immune-suppressive effect is mainly achieved by interleukin 10, prostaglandin E2 (PGE2), and TGF-*β* secretion of TAMs [144].

To summarize, macrophages in the tumor microenvironment are a major source of proangiogenic growth factors. Not only do they stimulate lymphangiogenesis through activation of endothelial cells, but they can also participate in this process by expressing LYVE-1 or becoming integral components of lymphatic vessels [146].

### 5.3. T-Cells

Recent research has led to a better understanding of the role of adaptive immune cells in the tumor microenvironment and first therapeutic options interfering with T-cell functions have successfully been US Food and Drug Administration (FDA) approved for antibody-based treatments in patients with advanced melanoma, for example, ipilimumab (see therapeutic approaches below).

A major attempt in development of immunological treatment strategies focuses on the identification of tumor-cell specific markers that may serve as therapy targets. Antigen recognition involves CD8+ T-cells recognizing tumor antigen [118]. Within the tumor microenvironment, two categories of CD8+ T-cells have been described. Some tumor tissues contain tumor-infiltrating T-cells, which secrete IFN-*γ*, whereas others lack T-cell infiltration and signs of inflammation. Whereas in the first group the tumor most likely inhibits the immune response, in the second group the immune system seems to ignore the ongoing tumor process (immune ignorance). The T-cell infiltrating phenotype has been shown for different types of cancer, including colorectal cancer [159, 160], renal cell carcinoma, melanoma, and ovarian cancer [161–164], and may have a positive prognostic value. A very good clinical outcome could be demonstrated for a high CD8+ T-cell to Fop3+ T_reg_ ratio in the ovarian cancer tumor microenvironment [165]. However, some melanomas still progress despite a T-cell infiltration, possibly related to a regress of the effectiveness of T-cells against tumor cells. This reduced effectiveness might be induced by the immunosuppressive tumor microenvironment [166]. The second group lacking tumor-infiltrating CD8+ T-cells was associated with an increased risk for metastasis into draining lymph nodes and decreased survival in dermal melanoma [161, 167].

Currently, the impact of T-cells on lymphangiogenesis and whether VEGF is involved in this context are mainly unknown. One study revealed that T-cells migrate responding to VEGF and that activated T-cells can express VEGF-R1 on their surface. Moreover, VEGF increased IL-10 secretion of these cells and might therefore direct chemotaxis and immune modulation of T-cells in tumor tissues [168].

### 5.4. B-Cells

Similar to T-cells, our knowledge on a potential role of B-cells in lymphangiogenesis is limited. Ruddell et al. reported B-cell accumulation in tumor draining lymph nodes, which induced lymphangiogenesis and increased lymphatic flow in E*μ*-*c-Myc* transgenic mice [169]. These mice exhibited increased lymphatic metastasis of lymphoma and melanoma [170]. Harrell et al. made similar observations. In a melanoma mouse model, B-cells were important for lymphangiogenesis and increased lymphatic flow through tumor draining lymph nodes [171]. However, the underlying mechanisms still need to be investigated.

## 6. Future Immunotherapeutic Strategies to Block Lymphangiogenesis and Prevent Lymphatic Metastasis

New therapeutic approaches have made it into clinical treatment to some extent. Although many of them are interfering with immune cell function, a secondary effect on lymphangiogenesis is expected, as immune activation induces lymphangiogenesis by different factors mentioned above. Below the latest therapeutic approaches or ideas are listed which may be used on a regular basis in the future to improve cancer treatment.

*VEGF Inhibiting Antibodies.* One of the first antibodies interfering with VEGF function was bevacizumab (Avastin), which was FDA approved for treatment of metastatic colorectal carcinoma [22]. Only a few studies have been performed with explicit focus on lymphangiogenesis. Sunitinib, a small molecule interfering with VEGF-R1 and VEGF-R2, PDGF α and PDGF *β*, KIT receptor, and Flt3 receptor [172], was FDA approved in 2006 and is currently used for the treatment of metastatic renal cell carcinoma and gastrointestinal stromal tumors [173]. A similar molecule is sorafenib, interfering with VEGF-R2 and VEGF-R3, PDGF receptor *β*, and c-KIT receptor [174], and is FDA approved for advanced renal cell carcinoma [175]. Studies in mice revealed that, by using RNA interference to inhibit VEGF-C expression, the lymphangiogenesis, the number of lymph node metastasis has been reduced and the survival prolonged [176]. Interfering with VEGF-R3 resulted in similar observations [177]. A VEGF-D blocking antibody reduced lymphatic metastasis in mice [23].

In human colorectal cancer, it has been shown that cyclooxygenase 2 (COX2) (involved in production of prostaglandins [178]) and VEGF-C are coexpressed [179]. In a mice lung cancer model celecoxib, a selective COX2 inhibitor reduced lymphangiogenesis and lymph node metastasis [180] indicating that VEGF expression and thereby lymphangiogenesis might be associated with prostaglandins.

Due to the increasing number of new therapeutics interfering with VEGF function, we refer to excellent reviews which address VEGF inhibitors in depth, for example, that by Takahashi [181].

*Programmed Death Ligand 1.* PDL1 is an immune regulator, expressed on APCs and in 20–50% on human cancer cells [133, 137]. Tumor-induced PDL1 inhibits T-cell function and induces immune tolerance but also apoptosis of T-cells [182]. In contrast, it induces the expansion of T_regs_ [183]. Therefore, blocking this ligand on the tumor cells and on antigen presenting cells improves tumor defense and T-cells with anticancer properties restore their effector function [184]. However, severe side effects have been reported when interfering with the immune system [185]. 

*Cancer Immunotherapy Using Dendritic Cells.* Targeting DCs and performance of an adoptive transfer, for example, with antigen loaded DCs, may improve immunotherapy in the future. There are different ways to vaccine DCs by using tumor lysate, viral vectors, DNA plasmids, or antigen peptides [186]. The optimized vaccination and administration approaches (intralymphatic, intravenous, or intradermal, etc.) are subject of ongoing research to improve clinical outcomes [186]. Furthermore, the procedure is restricted by the DC maturation state and dose finding [186]. It is possible that DCs in vivo might become suppressive DCs, thus counteracting antitumor immune responses. Despite its pros and cons, DC vaccination is a promising field for future improvements in cancer therapy. 

*Genetically Modified Autologous T-Cells.* Lately, there were first reports of patients who have been treated with an adoptive transfer of genetically modified autologous T-cells, which could improve certain B-cell malignancies [187, 188] or chronic lymphoid leukemia [189]. Thereby the T-cell antigen receptor was modified to target CD19 (expressed on B-cells) and a T-cell signaling molecule. First cases treated with these T-cells revealed a complete remission, although accompanied by adverse events during treatment [187–189]. Taken together, these first studies are showing promising results from autologous T-cell transfers and might improve cancer treatment in the future. 

*Anti-CTLA4 Antibodies.* In physiological conditions, T-cells are stimulated via CD28, which interacts with B7.1 and B7.2 on dendritic cells. Besides the “on button” CD28, T-cells express CTLA4, which can be regarded as “off button.” CTLA4 serves as a coinhibitor on activated T-cells to regulate their immune response [183]. Anti-CTLA4 antibodies such as ipilimumab are immune modulatory biologics and are regarded as a milestone in the treatment of metastatic melanoma [183]. Ipilimumab was FDA approved in 2011. It is able to block the major inhibitor of activated T-cells CTLA4 and blocks the interaction to its ligand B7.1 and B7.2 expressed on antigen presenting cells [190]. T-cells are thereby effectively and long-term activated to fight against tumor cells. However, immune modulatory biologics may have severe side effects, due to excessive and autoaggressive effects of the immune system [191]. CTLA4 deficient mice die early as a result of an uncontrolled lymphocyte proliferation that leads to multiorgan destruction [192].

Interfering with CTLA4 can also induce immune suppressive and immune tolerance: the antibody CTLA4-Ig-RFP occupies the B7.1 and B7.2 receptor on DCs and thereby blocks its interaction with CD28 [193]. 

*CCR7-CCL19/21.* Interfering with the CCL21-CCR7 axis to reduce immune cell or tumor cell migration has been tried in different approaches. Antagonists of CCL21 seem to prevent the development of chronic graft versus host disease [194] or reduced allergic conjunctivitis by blocking CCR7 in mice [195]. Obstructing CCR7 expression at mRNA level in a murine tumor model inhibited lymph node metastasis and lymphangiogenesis [196]. Pretreatment with an allogenic melanoma-derived cell lysate was capable of upregulating CCR7 expression on therapeutic human tumor presenting DCs and inducing migration to the lymph node [197]. This knowledge might be used for future improvement of immunotherapy. However, all studies interfering with the CCR7 axis in humans to treat cancer and metastasis are still in very early stages.

## 7. Conclusions

Lymphangiogenesis is a very early step in lymphatic metastasis. It is regulated and promoted not only by the tumor cells themselves, but also by cells of the tumor microenvironment, including cancer-associated fibroblasts, mesenchymal stem cells, dendritic cells, or macrophages. Even the extracellular matrix as well as cytokines and growth factors are involved in the process of lymphangiogenesis and metastasis. Many mechanisms behind lymphangiogenesis in the tumor microenvironmental crosstalk are still incompletely understood. A better insight of the underlying mechanisms might improve future therapeutics to reduce lymphatic spread of cancer cells to the draining lymph nodes in order to increase the survival of cancer patients. A personalized and thereby optimized therapy interfering with the affected parts of the tumor microenvironment is a promising approach for future treatment of lymphatic metastasis and thus tumor related death.
